# Periodontitis-Related Knowledge and Its Relationship with Oral Health Behavior among Adult Patients Seeking Professional Periodontal Care

**DOI:** 10.3390/jcm11061517

**Published:** 2022-03-10

**Authors:** Ewa Dolińska, Robert Milewski, Maria Julia Pietruska, Katarzyna Gumińska, Natalia Prysak, Tomasz Tarasewicz, Maciej Janica, Małgorzata Pietruska

**Affiliations:** 1 Department of Periodontal and Oral Mucosa Diseases, Medical University of Bialystok, ul. Waszyngtona 13, 15-269 Bialystok, Poland; mpietruska@wp.pl; 2 Department of Statistics and Medical Informatics, Medical University of Bialystok, ul. Szpitalna 37, 15-295 Bialystok, Poland; robert.milewski@umb.edu.pl; 3 Student’s Research Group, Department of Periodontal and Oral Mucosa Diseases, Medical University of Bialystok, ul. Waszyngtona 13, 15-269 Bialystok, Poland; maria.pietruska@gmail.com (M.J.P.); k.guminska@wp.pl (K.G.); natalia.prysak@gmail.com (N.P.); tarasewiczt@gmail.com (T.T.); 4 Student’s Research Group, Department of Statistics and Medical Informatics, Medical University of Bialystok, ul. Szpitalna 37, 15-295 Bialystok, Poland; janmac1e@gmail.com

**Keywords:** periodontitis, global health, current pathophysiological understanding of periodontitis, risk factors, modulators linking periodontitis and systemic diseases, oral hygiene, questionnaire study

## Abstract

Background: Periodontitis is a chronic inflammatory disease that not only damages the stomatognathic system, but may also adversely influence other systems and organs. Patients with low oral health literacy levels are more prone to gingivitis/periodontitis and have a more severe disease course. Methods: A written questionnaire was carried out to assess the knowledge of patients of the Outpatient Clinic of Department of Periodontal and Oral Mucosa Diseases, Medical University of Bialystok, Poland. The questions concerned knowledge regarding the causes of periodontal disease, its risk factors, and the connection between periodontal disease and general health status. To analyze the population, patients were divided according to gender, age and if they were first-time or regular outpatients. Results: Written questionnaires were completed by a total of 302 patients. In the studied population, we noted knowledge deficits, particularly related to weaker periodontal disease risk factors (stress, diabetes, osteoporosis, obesity) and the genetic factor, which is the determinant of periodontitis. The patients’ awareness of the role of plaque bacteria and the effect of smoking on the periodontium was at a relatively high level. The respondents were also aware of the impact of periodontal disease on general health as well as the role of oral hygiene in preventing the disease. At the same time, few of them (26%) used interdental brushes or an irrigator (8%). Conclusions: We demonstrated that patients have an insufficient level of knowledge related to risk factors as well as the prevention of periodontal disease. Awareness of the extent of oral health literacy among patients will help to identify key issues connected with health education interventions

## 1. Introduction

Periodontitis is a chronic inflammatory disease leading to bone and soft tissue destruction and, consequently, tooth loss. After dental caries, it is the major cause of tooth loss in adults [1]. Moreover, it is also the 11th most common disease in the world, and is more prevalent than cardiovascular diseases [2,3]. Severe forms of periodontitis may affect 10% of the adult population worldwide. The incidence of periodontal disease increases with age and rises rapidly in people aged 50–60 years. The proportion of people with periodontitis is expected to increase further as the population ages [4]. Despite efforts to improve oral health in recent years, periodontitis remains widespread and is a significant public health issue [5]. The World Health Organization (WHO) highlights that oral diseases (including periodontitis) are an important population problem due to their connections with other chronic diseases such as cardiovascular disease, diabetes and cancer, as well as their strong impact on people’s well-being and the high economic costs generated by treating these conditions [6]. Therefore, periodontal disease prevention should be approached from a new socio-economic perspective.

The prevention of diseases significantly determines an individual’s health and enables a considerable reduction in treatment-related costs. The patient’s engagement in oral health care correlates with their level of knowledge and health literacy (HL), defined as the ability to obtain, process, and use information to make appropriate decisions with an impact on one’s health. Patients with a low level of health literacy are less likely to adhere to the prescribed treatment, skip follow-up appointments, and apply a limited range of prophylaxis, and are more likely to suffer from general illnesses [7,8,9]. The same mechanism is crucial in terms of oral health literacy (OHL). Unfortunately, a limited ability to understand basic health information is common among adults and might have a significantly negative effect on the achievement of better results in maintaining sufficient oral hygiene [10]. Patients with low OHL levels are more prone to gingivitis/periodontitis and experience a more severe disease course. An increase in OHL level is correlated with undertaking preventive measures, following medical advice and an improvement in patients’ quality of life [7,8].

Periodontitis is a multifactorial disease affected by genetic and environmental risk factors, which may be divided into determinants (age, gender, ethnicity, gene polymorphisms) and acquired factors: environmental and behavioral (specific bacterial flora, smoking, stress, diabetes, obesity, osteoporosis, or socio-economic status) [11,12,13]. The development of periodontal disease is generally determined by biofilm accumulation, but the presence of other factors is individually responsible for one’s susceptibility or resistance to the disease. Reducing the influence of modifiable risk factors may alter the effectiveness of the prevention and treatment of periodontal disease.

Periodontitis not only damages the stomatognathic system, but also affects the chewing function and phonetics, and may adversely influence other systems and organs. Correlations between periodontitis and general diseases have been well documented and described since the 1990s. At present, there is a separate field of knowledge called “periodontal medicine” that evaluates the above-mentioned mechanisms [14,15,16]. Evidence supporting the link between periodontal disease and systemic diseases was discussed at the Joint EFP/AAP Workshop on Periodontitis and Systemic Diseases in 2012. Researchers from Europe and the USA mainly focused on the most thoroughly described associations of periodontal disease with diabetes, pregnancy complications and cardiovascular diseases. It was concluded that periodontal disease leads to a bacterial load, which results in a significant overall immune system response. This is likely to directly and indirectly affect the pathophysiology of general diseases [17,18,19]. As an example, both periodontitis and diabetes have an inflammatory basis and are linked together by different biochemical and metabolic interactions. Poorly controlled diabetes can increase the risk of periodontal disease, and periodontitis can adversely affect glycemic control mechanisms and increase the risk of diabetes complications [20]. It has been suggested that periodontal therapy may improve insulin sensitivity by reducing peripheral inflammatory cytokine levels. An improvement in glycemic status, defined as a reduction in glycated hemoglobin (HbA1c) was demonstrated in diabetic patients suffering from periodontitis [21,22]. There is also more evidence that periodontal therapy decreases plasma reactive oxygen species (ROMs), which are indicators of systemic oxidative stress [23].

The most widely reported associations in the literature on the subject are links between periodontitis and diabetes [20], cardiovascular disease [24], pregnancy and perinatal complications [25], obesity and metabolic syndrome [26], as well as rheumatoid arthritis [27], cancer [28], respiratory diseases [29], Alzheimer’s disease [30] and other cognitive disorders [16]. This knowledge is available to professionals but is not always available to a wider audience, including patients.

The aim of our study was to assess the level of patient knowledge regarding the causes of periodontal disease, its risk factors and the connection between periodontal disease and general health status in different age groups. We also evaluated patients’ health-promoting behaviors concerning oral hygiene.

## 2. Materials and Methods

### 2.1. General Methodology and a Questionnaire

We assessed the knowledge of periodontal disease in the patient population at the Outpatient Clinic of Department of Periodontal and Oral Mucosa Diseases at the Medical University of Bialystok, Poland, in the period from April 2016 to November 2017.

Patients specified their age, gender and whether it was their first visit to the Outpatient Clinic.The main questions included in the questionnaire were connected to the following:

- Causes of periodontal disease;
- Risk factors of periodontal disease;
- Impact of periodontal disease on general health;
- Pro-health behaviors of patients, aimed at prevention of periodontal disease.

Furthermore, the patients were asked which dental hygiene devices they used. We assumed that the use of interdental hygiene utensils (interdental brushes, dental floss) was a positive, health-promoting behavior resulting from awareness of periodontal disease prevention.

Additional questions pertained to:

- The frequency of tooth brushing;
- The use of a manual or mechanical toothbrush;
- The use of dental floss, interdental brushes, single-tuft brushes toothpicks or irrigators;
- The use of additional pharmacological agents such as mouthwashes, ointments, gels, breath fresheners and herbal remedies.

The questionnaire administered to participants is reported in Table 1.

The research was conducted in accordance with the Declaration of Helsinki, and approval was obtained from the local bioethics committee (R-I-002/80/2016). Subjects filled in the anonymous questionnaire form voluntarily, which was considered equivalent to consenting to participate in the study.

### 2.2. Statistical Analysis

For descriptive purposes, we first analyzed the number of correct answers to each question. If the number of the respondents who answered a question correctly did not exceed 80%, we concluded that the given group had insufficient knowledge about the topic included in the given question. We analyzed answers to each question according to the gender and age of the participants. The patients were also divided into two groups based on whether they were first-time patients or on a subsequent visit to the Clinic.

In the statistical analysis, the Chi-square test of independence was used to check the relationship between qualitative characteristics. Statistical significance was established at *p* < 0.05. Calculations were made by means of a Statistica 13.3 package from TIBCO Software Inc. (Palo Alto, CA, USA).

## 3. Results

### 3.1. Description of the Studied Group

Written questionnaires were completed by a total of 302 patients, including 180 women and 105 men (gender of 17 participants was not documented). The majority of patients completing the questionnaire were former outpatients (*n* = 189), and 36% were first-time patients. Smokers constituted 15% of the surveyed group. The patients were divided into the following age groups: 21–30 years (10% of the respondents), 31–40 years (15%), 41–50 years (22%), 51–60 (26.5%), 61–70 (17%) and 71–80 (7%) (the age of the remaining percentage of patients was not documented). The characteristics of the study group according to age, gender and status in the outpatient clinic are presented in Table 2. As some questionnaire variables were incomplete, the total numbers for some of the data collected in the questionnaire differ.

The response rate to the questionnaire was 15.2% (out of 1988 patients who attended an appointment at the Outpatient Clinic in the period from April 2016 to November 2017, 302 people completed the questionnaire).

### 3.2. Knowledge Regarding the Causes of Periodontal Disease

Participants in our study answered seven questions about the risk factors for periodontal disease. The questions concerned bacteria forming the dental plaque, genetic factors, smoking, stress, diabetes, osteoporosis, and obesity. The involvement of bacteria in the etiology of periodontal disease was confirmed by 81% of the survey respondents. Insufficient knowledge on the subject (<80% of correct answers) was mostly demonstrated by men (77% correct answers), and those in the 41–50 age group (70%). The link between genetic factors and the occurrence of periodontal disease was reported by 61% of the patients. Lack of knowledge about this determinant of periodontal disease was evident in all age and gender groups, regardless of whether the patient was an outpatient or in the clinic for the first time. Tobacco smoking was associated with periodontal disease by 85% of respondents. Only patients aged 71–80 years showed insufficient knowledge (77% of correct answers) in this field. Significant knowledge deficiencies were noted for weaker risk factors. Stress was confirmed to be related to periodontal disease by 61% of respondents, diabetes by 64%, osteoporosis by 62% and obesity by only 39% of the surveyed patients. Stress and osteoporosis were statistically significantly more often reported as risk factors leading to periodontal disease by women. A detailed analysis of the respondents’ knowledge of periodontal disease risk factors is presented in Table 3.

### 3.3. Knowledge of Risk of Other Diseases Associated with Periodontal Disease

An overwhelming number of respondents (89%) answered the question “Do you think that periodontal disease affects your overall health?” with “yes”. The highest percentage of affirmative responses was obtained in the 41–50 age group (93%). A detailed distribution of the responses is presented in Table 4.

### 3.4. Knowledge Regarding Prevention of Periodontal Disease

To assess the awareness regarding periodontal disease prevention, the participants in our study answered the question “Do you think that inadequate oral hygiene may lead to the occurrence of periodontal disease?” More than 90% of the total number of respondents answered affirmatively, and in the youngest age groups (21–30 years, 31–40 years), a positive answer was given by 100% of the respondents. A detailed analysis of the responses is presented in Table 5. Simultaneously, the health-promoting behavior was assessed by asking participants about their daily hygiene habits as well as the use of dental hygiene devices and additional pharmacological agents. The majority of respondents (90%) admitted that they brushed their teeth at least twice a day. A manual toothbrush was used by 78% of the participants. Flossing was reported by 64% of the patients (significantly more women than men, p = 0.0008), and interdental brushes were used by only 26% of the respondents (significantly more women, p = 0.03). At the same time, 24% of the participants used toothpicks and only 8% used an irrigator. Table 6 contains a detailed analysis of health-promoting activities, separated by gender, age, and whether the patient was visiting the clinic for the first time or was a regular outpatient.

## 4. Discussion

The survey aimed to evaluate the knowledge of patients seeking periodontal care and their involvement in their daily hygiene regimen. In the studied population, we noted knowledge deficits, particularly related to weaker periodontal disease risk factors (stress, diabetes, osteoporosis, obesity) and the genetic factor, which is the determinant of periodontitis. The patients’ awareness of the role of plaque bacteria and the effect of smoking on the periodontium was at a relatively high level. The respondents were also aware of the impact of periodontal disease on general health as well as the role of oral hygiene in preventing the disease. At the same time, few of them (26%) used interdental brushes or an irrigator (8%). Supportive pharmacological agents were more popular. Oral rinses were used by 66% of the participants. Knowledge deficits were most visible in the oldest age group (71–80 years). These findings are consistent with the reports of other researchers, who suggested that knowledge deficits are associated with lower education levels and the age of patients [31,32]. However, according to the analysis of the collected survey data, patients of all age groups need education in the discussed field, not only in terms of the causes of periodontal disease but also its prevention at home.

Home oral hygiene involves using a toothbrush, dental floss, toothpicks, and other devices to remove plaque and food particles from the surface of the teeth. Individual oral hygiene is often considered a key factor in controlling periodontal disease, thus providing an enormous benefit to public health. Despite the lack of direct evidence in the form of randomized clinical trials to confirm the relationship between oral hygiene and periodontal disease, maintaining optimal oral hygiene is a fundamental principle of periodontal disease prophylaxis [33]. Home hygiene, causal treatment, and maintenance therapy are keystones in disease prevention [34], while neglecting oral hygiene leads to the accumulation of plaque, dental calculus, and development of gingivitis [35]. Brushing teeth twice a day with fluoride toothpaste is a basic hygiene procedure performed in developed countries. In the studied population, only 7% of women and 13% of men reported brushing their teeth only once or less frequently per day. This was also the case for 12% of first-time patients and 8% of regular outpatients at the Clinic. The percentage of flossers was also high: 64%. Using dental floss was reported by 50% of men and 70% of women, and interdental brushes by 19% of men and 31% of women. Toothpicks were most popular among those over 40 years of age. As many as 92% of the respondents treated previously at the Outpatient Clinic brushed their teeth twice or more times a day, 66% of whom used floss, while 29% used interdental brushes and 8% reported using an irrigator. The percentage in each case was higher than for patients waiting for their first periodontal visit.

A survey on oral hygiene in the entire Polish population was conducted by Górska and Górski in 2018 (survey in 10 cities). The use of dental floss was reported by 57% of the respondents, interdental brushes by 12%, an irrigator by 8%, and mouthwash by 67% [36]. These results are similar to ours, particularly in terms of the use of an irrigator and mouthwashes. Fewer respondents reported using dental floss and interdental brushes. This is largely related to the fact that the survey was focused on the general population rather than those seeking professional periodontal care. Self-reported oral hygiene was much worse in the Italian population, in which only 23.5% of respondents brushed their teeth twice or more times a day, and daily flossing was reported by 13.3% of people. The recommendation of the Italian authorities to brush one’s teeth regularly twice a day and attend check-up visits to the dentist once a year was met by only 12% of the respondents [37].

In the Lithuanian population, over a 20-year time period (1994–2014), the 20–64 age group demonstrated an improvement in the frequency of oral hygiene procedures. There, the percentage of men brushing their teeth at least twice a day increased from 15% to 32% and, in women, from 33% to 59% [38]. Portuguese self-reported oral hygiene surveys reached similar values to the European average. In the examined Portuguese population, 73% of the respondents brushed their teeth at least twice a day (78% of women and 69% of men). Flossing was reported by 29% of women and 18% of men [39].

Effective plaque removal at home plays a key role in the prevention and treatment of periodontal disease. Cleaning the interdental spaces is also very important [40]. In this case, the most effective hygienic aids are interdental space brushes as they remove more plaque than floss or toothpicks [41]. In periodontal patients, these devices should be the first choice for interdental cleaning. The use of interdental cleaning utensils can be considered an indicator of active knowledge of periodontal disease prevention [42].

Individually tailored educational programs related to improvements in oral hygiene may encourage patients to clean the interdental spaces more frequently and maintain a high level of commitment and behavior change. Such programs improve long-term adherence to oral hygiene in periodontal treatment [43]. Optimal oral hygiene leads not only to a change in periodontal indices but also influences the general condition of patients with diabetes and hypertension. Four oral hygiene sessions with a dental hygienist were sufficient to maintain stable blood pressures and significantly lower glycosylated hemoglobin levels at the fourth session [44]. It was proven that a higher level of knowledge about periodontal disease, its pathogenesis, and consequences led to internal motivation and an improvement in oral hygiene, especially in the interdental spaces [45].

When analyzing the results of the presented experiment, the limitations of our survey should also be considered. The study group consisted of patients seeking professional periodontal help at the Outpatient Clinic at the Medical University. The knowledge of this group of patients cannot be compared to the entire Polish population, including those without symptoms of periodontal disease. Another limitation of our work was the size of the assessed group. Despite the fact that our survey lasted over a year, only 302 people decided to complete our questionnaire. The likely reason for this was the need to fill out multiple forms before the periodontal visit, which is particularly cumbersome for the elderly. In our survey, we did not ask about education and socioeconomic status. Although additional questions could provide new information and dependencies, we wanted to avoid the survey being too long. We did not correlate periodontal status with the level of patients’ knowledge, which allowed the research to remain anonymous and a larger group of respondents to be gathered. 

It is also important to recognize that patients’ knowledge is not the only factor affecting oral health; other aspects described by health behavior models are equally important in motivating patients to change their hygiene habits. These evidence-based psychological models are connected, inter alia, with self-efficacy, motivation, counselling, decision balance (relationship of perceived benefits and behavioral barriers), perceived susceptibility, and normative beliefs [46,47,48]. Knowledge is only a prerequisite, but it is necessary to improve patients’ health-seeking behaviors.

## 5. Conclusions

In our work, we demonstrated that patients have an insufficient level of knowledge related to the risk factors and prevention of periodontal disease, especially through effective interdental cleaning. The collected data indicate the need for further education on periodontal disease among patients attending the Outpatient Clinic for Periodontal Diseases of the Medical University of Bialystok and in the general Polish population. Awareness of the extent of OHL among patients will enable the identification of key issues connected with oral health education interventions. The effective education of patients should result in more successful prevention and treatment of periodontal diseases.

## Figures and Tables

**Table 1:** Questionnaire administered to participants.

1.	Gender					Male	Female
2.	Age	21–30	31–40	41–50	51–60	61–70	71–80
3.	Is it your first visit in the Outpatient Clinic of Department of Periodontal and Oral Mucosa Diseases?					Yes	No
4.	Do you think that oral bacteria contribute to the presence of periodontal disease?					Yes	No
5.	Do you think hereditary factors contribute to the presence of periodontal disease?					Yes	No
6.	Do you think that smoking contributes to the presence of periodontal disease?					Yes	No
7.	Are you a smoker?					Yes	No
8.	Do you think that stress affects the presence of periodontal disease?					Yes	No
9.	Do you think that diabetes contributes to the presence of periodontal disease?					Yes	No
10.	Do you think that osteoporosis contributes to the presence of periodontal disease?					Yes	No
11.	Do you think that obesity contributes to the presence of periodontal disease?					Yes	No
12.	Do you think that periodontal disease affects your overall health?					Yes	No
13.	Do you think that inadequate oral hygiene affects the presence of periodontal disease?					Yes	No
14.	How many times a day do you brush your teeth?					0	
						1	
						2	
						More than 2	
15.	What kind of brush do you use?					Manual toothbrush	
						Mechanical toothbrush	
16.	Do you regularly use any additional dental devices?					No	
						Dental floss	
						Interdental brushes	
						Single-tuft brushes	
						Toothpicks	
						Irrigator	
17.	Do you use any additional pharmacological agents?					No	
						Mouthwashes	
						Oral gels	
						Oral ointments	
						Breath fresheners	
						Herbal remedies	

**Table 2:** Sample characteristics: number of patients in the respective groups. As some questionnaires variables were incomplete, the total numbers for some of the collected in the questionnaire data differs.

	Total	Man	Women	First Time Patient	Regular Patient
Total	302	105	180	110	189
21–30	31	10	19	15	16
31–40	45	18	25	25	20
41–50	67	27	39	24	43
51–60	80	23	53	25	53
61–70	52	16	31	11	41
71–80	22	10	11	8	14

**Table 3:** Patient’s knowledge about the influence of risk factors on the occurrence of periodontal disease.

	Age Group						Chi-Square	*p*
Bacteria	21–30 (*n* = 26)	31–40 (*n* = 43)	41–50 (*n* = 46)	51–60 (*n* = 67)	61–70 (*n* = 43)	71–80 (*n* = 18)	12.7	*p* = 0.03
	84%	96%	70%	84%	83%	82%		
	Male (*n* = 81)			Female (*n* = 152)			2.7	NS
	77%			85%				
	First time patients (*n* = 91)			Regular patients (*n* = 152)			0.2	NS
	83%			81%				
Genetics	Age group							
	21–30 (*n* = 23)	31–40 (*n* = 32)	41–50 (*n* = 44)	51–60 (*n* = 49)	61–70 (*n* = 22)	71–80 (*n* = 11)	14.9	*p* = 0.01
	74%	73%	68%	63%	42%	50%		
	Male (*n* = 61)			Female (*n* = 112)			0.4	NS
	59%			63%				
	First time patients (*n* = 70)			Regular patients (*n* = 114)			0.2	NS
	64%			62%				
Smoking	Age group							
	21–30 (*n* = 26)	31–40 (*n* = 41)	41–50 (*n* = 63)	51–60 (*n* = 64)	61–70 (*n* = 43)	71–80 (*n* = 17)	7.9	NS
	84%	91%	94%	81%	84%	77%		
	Male (*n* = 90)			Female (*n* = 154)			0.01	NS
	87%			86%				
	First time patients (*n* = 96)			Regular patients (*n* = 160)			0.5	NS
	88%			85%				
Stress	Age group							
	21–30 (*n* = 19)	31–40 (*n* = 27)	41–50 (*n* = 46)	51–60 (*n* = 42)	61–70 (*n* = 37)	71–80 (*n* = 10)	7.8	NS
	61%	60%	69%	54%	73%	48%		
	Male (*n* = 54)			Female (*n* = 121)			7.7	*p* = 0.006
	51%			68%				
	First time patients (*n* = 65)			Regular patients (*n* = 118)			0.3	NS
	60%			63%				
Diabetes	Age group							
	21–30 (*n* = 23)	31–40 (*n* = 33)	41–50 (*n* = 44)	51–60 (*n* = 48)	61–70 (*n* = 29)	71–80 (*n* = 10)	8.0	NS
	74%	73%	66%	65%	56%	45%		
	Male (*n* = 66)			Female (*n* = 112)			0.005	NS
	64%			64%				
	First time patients (*n* = 75)			Regular patients (*n* = 114)			1.1	NS
	69%			62%				
Osteoporosis	Age group							
	21–30 (*n* = 18)	31–40 (*n* = 31)	41–50 (*n* = 43)	51–60 (*n* = 49)	61–70 (*n* = 34)	71–80 (*n* = 9)	5.6	NS
	60%	69%	64%	64%	65%	41%		
	Male (*n* = 54)			Female (*n* = 120)			7.8	*p* = 0.005
	51%			68%				
	First time patients (*n* = 73)			Regular patients (*n* = 112)			2.0	NS
	68%			60%				
Obesity	Age group							
	21–30 (*n* = 15)	31–40 (*n* = 14)	41–50 (*n* = 27)	51–60 (*n* = 27)	61–70 (*n* = 25)	71–80 (*n* = 6)	6.0	NS
	48%	32%	41%	36%	50%	29%		
	Male (*n* = 41)			Female (*n* = 70)			0.001	NS
	40%			40%				
	First time patients (*n* = 39)			Regular patients (*n* = 76)			0.6	NS
	37%			41%				

NS: non significant.

**Table 4:** Patients’ knowledge about the influence of periodontitis on general health status.

Age Group						Chi-Square	*p*
21–30 (*n* = 28)	31–40 (*n* = 41)	41–50 (*n* = 62)	51–60 (*n* = 66)	61–70 (*n* = 47)	71–80 (*n* = 20)	1.7	NS
90%	91%	93%	87%	92%	91%		
Male (*n* = 95)			Female (*n* = 159)			0.001	NS
90%			90%				
First time patients (*n* = 97)			Regular patients (*n* = 169)			0.09	NS
90%			91%				

NS: non significant.

**Table 5:** Patient’s knowledge about the role of optimal oral hygiene in prevention of periodontitis (Do you think that inadequate oral hygiene affects the presence of periodontal diseases?).

Age Group						Chi-Square	*p*
21–30 (*n* = 31)	31–40 (*n* = 43)	41–50 (*n* = 64)	51–60 (*n* = 73)	61–70 (*n* = 46)	71–80 (*n* = 19)	10.4	NS
100%	100%	97%	92%	90%	86%		
Male (*n* = 100)			Female (*n* = 165)			1.9	NS
97%			93%				
First time patients (*n* = 100)			Regular patients (*n* = 177)			1.3	NS
93%			96%				

NS: non significant.

**Table 6:** Self-reported oral hygiene in different age, gender and outpatient clinic status patients (data as a percentage).

Oral Hygiene Practice: Percentage of Participants	Gender			Age Group							Patients Status		
	F	M	*p*	21–30	31–40	41–50	51–60	61–70	71–80	*p*	First Time	Regular	*p*
Brush once a day	7	13	NS	10	4	12	10	12	4	NS	12	8	*p* = 0.02
Brush twice a day	67	71		68	78	64	66	65	64		73	63	
Brush more than twice a day	26	16		22	18	24	24	24	32		15	29	
Manual toothbrush	75	84	NS	65	65	66	86	94	96	*p* = 0.0003	80	77	NS
Mechanical toothbrush	25	16		35	35	34	14	6	4		20	23	
Dental floss	70	50	*p* = 0.0008	65	76	73	63	59	36	*p* = 0.02	61	66	NS
Interdental toothbrushes	31	19	*p* = 0.03	19	33	23	28	31	18	NS	21	29	NS
Toothpics	24	24	NS	6	16	28	34	22	23	*p* = 0.03	24	23	NS
Irrigator	10	5	NS	6	9	12	10	2	0	NS	6	8	NS
Mouthwashes	66	66	NS	84	80	66	62	63	45	*p* = 0.02	61	70	NS
Oral gels	11	5	NS	3	7	12	10	8	9	NS	9	8	NS
Oral ointments	7	4	NS	3	2	4	8	10	9	NS	5	6	NS
Breath fresheners	9	6	NS	0	9	9	11	2	10	NS	7	7	NS
Herbal remedies	18	12	NS	3	22	13	19	20	14	NS	18	16	NS

NS: non significant, *p*: Chi square test.

## Data Availability

The datasets used and/or analyzed during the current study are available from the corresponding author on reasonable request.
